# The embryo-oil drop assembly: the timing and morphology of a critical event for fish early-life history survival

**DOI:** 10.1038/s41598-024-57429-9

**Published:** 2024-03-22

**Authors:** Manuel Nande, Montse Pérez, Pablo Presa

**Affiliations:** 1 AQUACOV, Instituto Español de Oceanografía (CSIC-IEO), Centro Oceanográfico de Vigo, Vigo, Spain; 2 Laboratory of Marine Genetic Resources, ReXenMar-CIM-Universidade de Vigo, 36310 Vigo, Spain; 3 Present Address: CIIMAR-Interdisciplinary Centre of Marine and Environmental Research, University of Porto, Terminal de Cruzeiros de Leixões, Av., General Norton de Matos s/n, Porto, Portugal

**Keywords:** Egg buoyance, Egg gravity, Fish embryo, Marine fisheries, Oceanic environment, Yolk syncytial layer, Gastrulation, Marine biology, Developmental biology, Ecology, Climate sciences, Ocean sciences

## Abstract

Egg specific gravity is of relevance for fish recruitment since the ability to float influences egg and larvae development, dispersal and connectivity between fishing grounds. Using zootechnics, histological approaches, optical and electronic transmission microscopy, this study describes the morphogenetic mechanism of adhesion of the oil-drop covering layer (OCL) to the oil droplet (OD) in embryos of *Merluccius merluccius* under physical conditions reflecting the marine environment. The herein described primordial (p)OCL is a substructure of the inner yolk syncytial layer which contains egg organella aimed to mobilize lipidic reserves from the oil drop (OD) towards the embryo blood. It is shown that the timely OD-OCL assembly is a critical morphogenetic process for embryo and larvae survival. Such assembly depends on egg buoyance because of its influence on the embryo capacity to rotate within the perivitelline space. Therefore, oil droplet adhesion (ODA) eggs are capable to complete their development while oil droplet non-adhesion eggs (ODNA) dye soon after hatching. We show that gravity-dependent egg buoyance categories exhibit different ODA/ODNA ratios (0–77%) and that relationship diminishes under incubation systems such as sprayers, that do not assure a dynamic seawater surface mixing to avoid egg desiccation. As an adaptive trait, egg gravity strongly depends on oceanic properties such as current dynamics, turbulence, oxygen, rainfall, and salinity, whose rapid changes would likely challenge the sustainability of fisheries recruitment.

## Introduction

**Figure 1:**
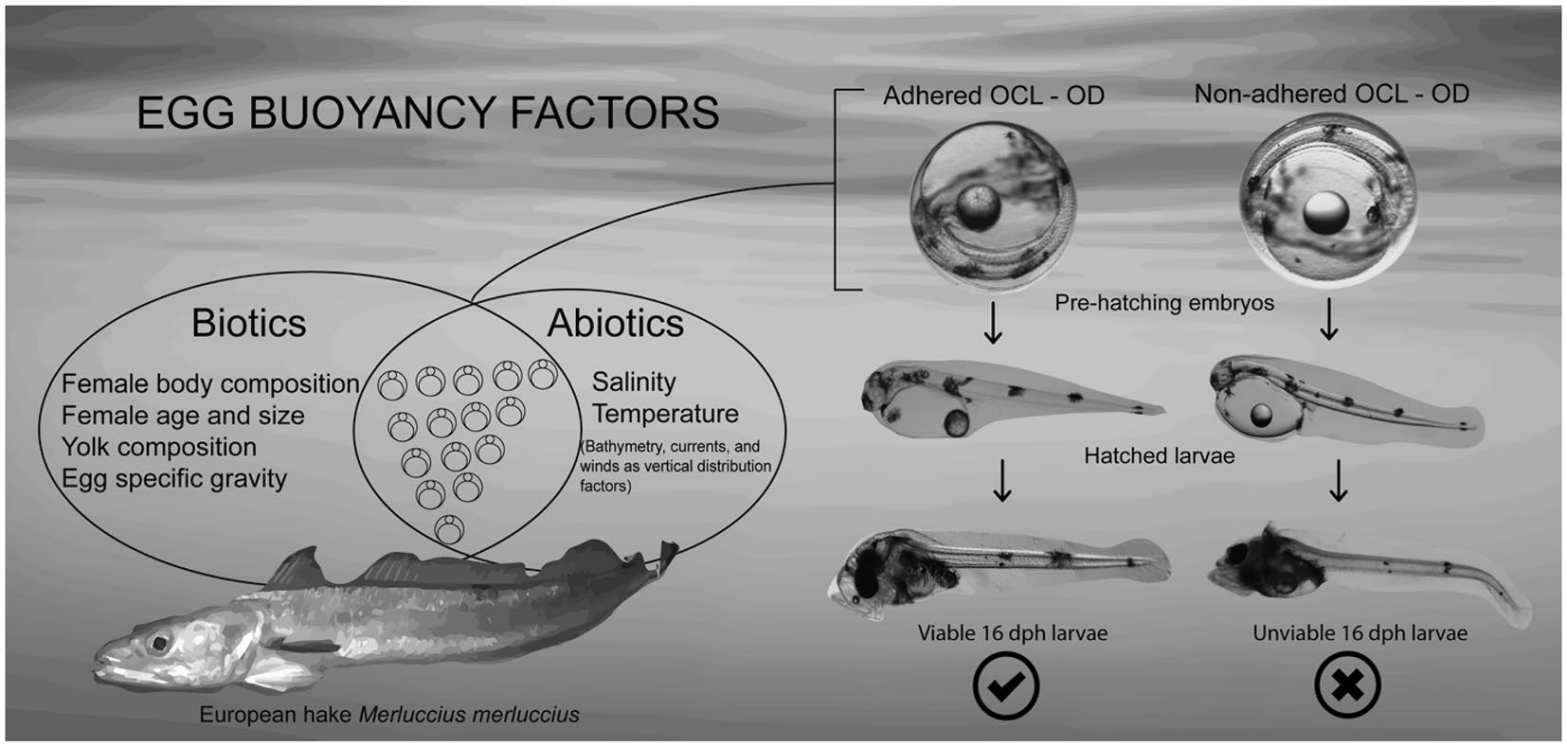
Conceptual illustration indicating known biotic and abiotic factors influencing buoyancy on European hake eggs (left panel). The correct interaction between the oil cover layer (OCL) and the oil droplet (OD) during embryonic development determines the formation of the OCL–OD complex, which plays a determinant role in larval viability (right panel). An incorrect OCL–OD embryonic assembly prevents the mobilization and assimilation of vital OD nutrient content by larvae.

The bathymetric distribution of marine fish eggs is determined by buoyancy, which results from the interaction between the physical environment factors such as salinity and temperature and the specific egg gravity^1^. The physical input is related with seawater properties affecting the vertical distribution of eggs, comprising density, viscosity, turbulence, and up/down-welling phenomena^1^. Egg gravity is a function of physiochemical properties such as chorion thickness, volume and yolk sac osmolarity^2^. Those structures are involved in the first egg hydration during oogenesis which determines a critical egg density at spawning^3^ as well as egg osmoregulation after fertilization^4^. Osmoregulation comprises a balance between seawater and internal free amino acids (FAAs) from yolk proteins^5,6^ and is regulated by vitelline membranes via channel aquaporins^3^ (Fig. 1).

Once the egg-own osmoregulatory mechanism is fully established after gastrulation, the density of the perivitelline space is at equilibrium with the surrounding environment whereas the density of the yolk–embryo changes during ontogeny^7,8^. The osmoregulatory balance determines a varying egg buoyance as reported to occur at most organization levels, i.e. per developmental stage, e.g. in Norwegian coastal cod^9^, Baltic cod^10,11^, polar cod^12^, or anchovies^13^, between spawns within female^2^, between females upon age and season^14^, within and between stocks, e.g. in anchoveta^15^ and the flounder^16^. Provided that a developmental success occurs at the spawn critical layer^17^, species-specific egg gravity and hydration regulation allow eggs to emerge from spawning areas to photic areas to complete larval development^18^. Such positioning in the seawater column involves a dynamics that is highly dependent on the local oceanography which affects eggs horizontal and vertical distribution.

In the organism, fish embryos and larvae obtain energy during their ontogenetic development first from the yolk sac [YS] and later from the oil droplet [OD]^19^. The resorption of yolk and droplet reserves in fish is afforded by the specialized structure termed yolk syncytial layer (YSL), periblast or vitelline syncytium, which separates and surrounds those two reservoirs and is reabsorbed once the YS becomes exhausted^20^. The formation of YSL was reported in *Ctenolabrus* spp.^21^, then described in *Serranus alranus*^22^ and more recently described in trout^23^, zebrafish^24^, *Fundulus heteroclitus*^25^, sea bass, sea bream and pike-perch^26^ and turbot^27^. During early development the YSL synthesizes lipoproteins as triggered by the apolipoprotein [apoE] gene^27,28^ that transfer lipids from YS reserves and OD via the endoplasmic reticulum and the Golgi apparatus to the perivitelline space and reach the embryo blood system^29^. The YSL uses YS and a large part of the OD droplet from hatching until the first days after mouth-opening. The OD is a key endotrophic structure comprised in the YS which contains primarily polar lipids as prominent components of fish eggs, but also types of non-polar lipids which are critical for a normal development^30,31^.

A well-known species regarding both its bathymetric distribution and embryogenesis is the European hake, *Merluccius merluccius* Linnaeus (1758)^32,33^, a key trophic organism inhabiting 30‒500 m in depth from the Northeast Atlantic Ocean (Iceland) and the Mediterranean Sea to the coast of Mauritania^34^. A workflow system for early hake feeding has been worked out^35^ based on the methodological work developed for the European hake in the last decade^33,36^.

Particularly, the adherence of the oil droplet (OD) to the yolk syncytial layer (YSL), termed here OD–YSL complex, has been one of the criteria used to evaluate the quality of spontaneous egg layers from the first captive hake broodstock. i.e. eggs with a non-adhered OD–YSL do not make use of endogenous reserves what determines the irremissible larvae death at 15‒20 days post-hatching (dph). This lack of OD-YSL assembly seriously limited the viability of larval cultures in the European hake^37^ as also reported in the congeneric hake *M. australis*, i.e. 31‒81% of non-assembled OD-YSL eggs^38^. Despite such OD-YSL complex plays a key developmental function, very little is known on the influence that internal imprinting (gravity, developmental stability, morphogenetic determinism) and environment (salinity, turbulence, oxygen, etc.) have on the successful assembly of de OD-YSL complex. It has been suggested that broodstock stress may affect the adequate absorption of the OD because of malformations in either the YSL or in the intestinal mucosa of larvae^27,39^.

As outlined above, the causation of oil droplet non-adhesion (ODNA) eggs remains unknown and has been potentially assigned to a YSL formation failure^38^. However, we hypothesized that ODNA eggs and their subsequent larval death could be due to a wrong bathymetric egg status preventing the formation of the OD-YSL complex, either because a suboptimal egg gravity and/or a suboptimal placement in the seawater layer. Tanking advantage that European hake eggs contain a single OD located at the vegetal pole as opposite to the blastodisc and the developing embryo, this study aimed to assess the ontogenic changes in OD-YSL formation until hatching. The dissection of hake embryogenesis allowed to address the relationship among forces determining the assembly of the OD-YSL complex during development, e.g. buoyancy, the morphogenetic dynamics of such single OD. This task was accomplished in vitro using microscopic characterization and the ultrastructural description of the oil covering layer primordium (pOCL) and the OD-YSL complex. By implementing in vitro experimental designs we also address the role of internal determinism (egg gravity) and some external forces (e.g. surface turbulence) on the OD-YSL assembly success and propose its putative causal morphogenetic mechanism.

## Results

### Embryonic development of the European hake

**Figure 2:**
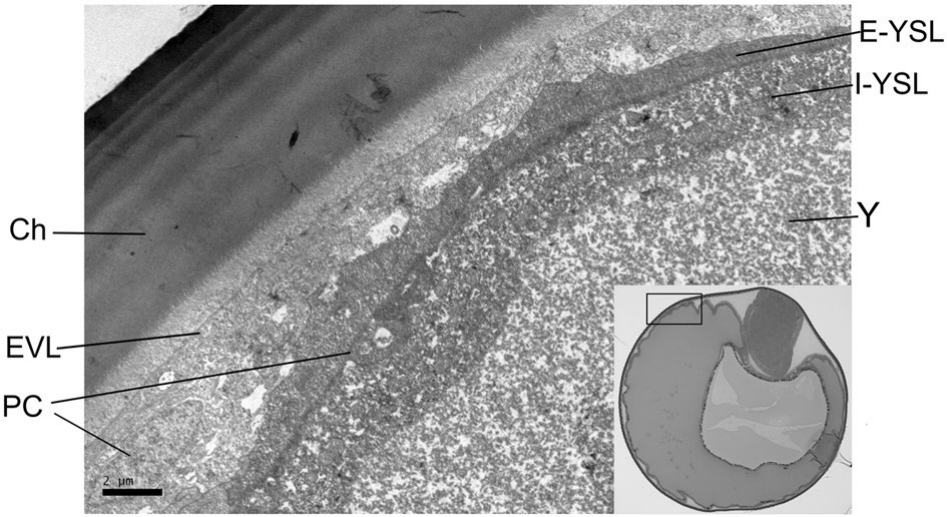
Ultrastructure of the cellular membrane at blastula (26 hpf, stage C, Fig. S1 1.8). The chorion (Ch) appears external to the pervitelline space, the syncytium and the yolk (Y). The syncytium comprises the enveloping layer (EVL), the external yolk syncytial layer (E-YSL), the internal yolk syncytial layer (I-YSL) and some primordial germ cells (PC).

Subsequent histological screening showed that the embryonic development of the European hake lasted 4 days at 14 °C. The zygote formed in the animal pole within the first hour post fertilization (hpf) (Fig. S1 1.1). The first division (2 blastomeres) occurred at 1 hpf (Fig. S1 1.2); four and eight blastomeres were formed at 4 hpf and 7 hpf, respectively (Fig. S1 1.3 and Fig. S1 1.4). Successive cleavages up to Morula stage were observed from 8 to 15 hpf (e.g. Fig. S1 1.5 and Fig. S1 1.6). Morula (stage B) and blastula (stage C) were patent at 18 hpf and 26 hpf, respectively (Fig. S1 1.7–1.9). The YSL was visible at cell stage B (Morula, 512-cell, Fig. S1 1.7). The anchorage between two layers of differentiated cells, i.e. the Enveloping Layer (EVL) and the Yolk Syncytial Layer (YSL) was observed since early gastrula (Fig. S1 1.8 and Fig. 2). Gastrula was observed at 31 hpf (stage D) when epiboly begun and fully covered embryo and yolk at 40 hpf (Fig. S1 1.10 and Fig. S1 1.11). The hake embryo was observed in the animal pole at 48 hpf (stage E) with the oil droplet (OD) on the vegetal pole (Fig. S1 1.12, ventral view). Embryo growth was patent at 54 hpf (stage F, Fig. S1 1.13), nearly occupied the entire egg perimeter by 76 hpf (stage G, Fig. S1 1.14) and hatched at 96 hpf (stage H, Fig. S1 1.15).

### Structure and ultrastructure of the OD–OCL assembly mechanism

Embryo at stage E was located at the animal pole along with the perivitelline space which was more prominent in head and tail, keeping this equilibrium position (ventral view in Fig. 3.1). The OD at stage E was positioned at the vegetal pole (apical view in Fig. 3.1). Eggs embedded in glutaraldehyde lost buoyancy due to dehydration but embryo maintained its location inside the egg despite being twisted manually into different angles. However, OD shifted upwards systematically due to its positive buoyancy. That phenomenon implied a free-floating OD in the yolk sac during stage E (Fig. 3.2), i.e. well before the oil covering layer (OCL) entrapped it fully (Figs. 3.3, 3.4, 4).

**Figure 3:**
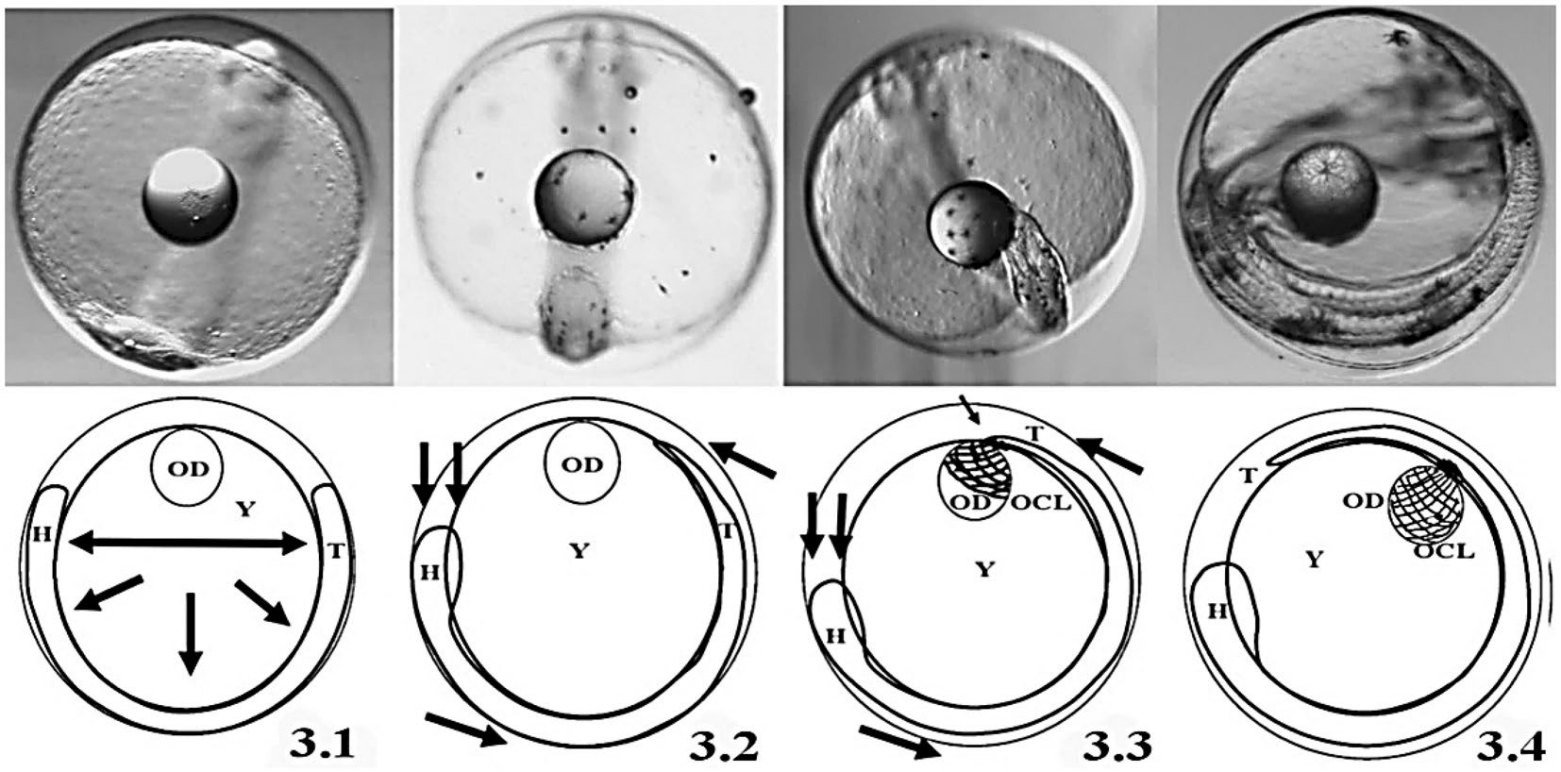
Morphogenetic mechanism involved in the transition from a floating oil droplet (OD) to its entrapping by the oil covering layer (OCL). (**3.1**) Developmental stage E (48 hpf); (**3.2**) Transition between stage E and stage F (54 hpf); (**3.3**) Developmental stage F; (**3.4**) Developmental stage G (76 hpf). *H* embryo head, *Y* yolk sac, *T* embryo tail.

**Figure 4:**
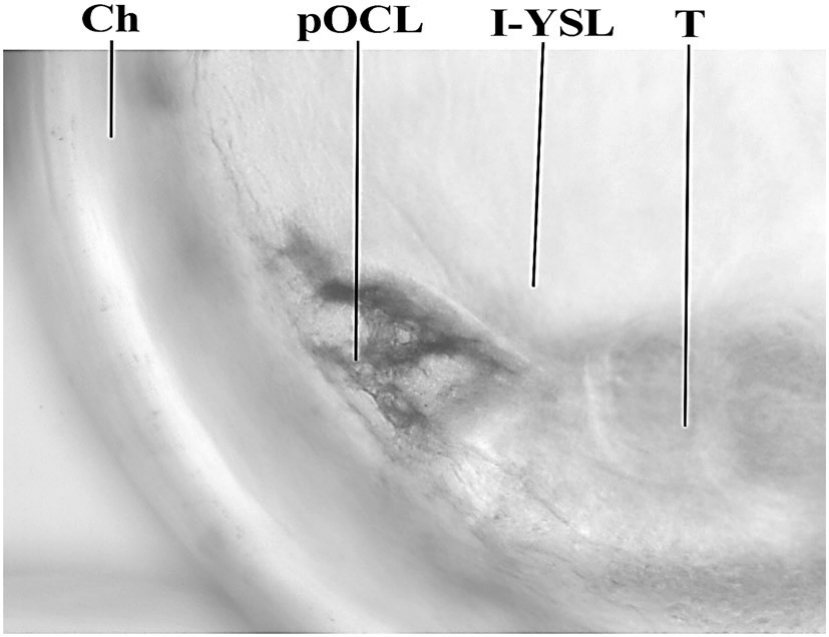
Microscopy showing the formation of the primordium oil cover layer (pOCL) after the inner yolk syncytial layer (I-YSL) near the embryo tail (T) during transition from stage E (48 hpf) to stage F (54 hpf). Ch, chorion. Observe the absence of the OD in the neighborhood of the pOCL primordium.

Embryo grew in length during the transition from stage E to stage F and occupied ~ 70% of the egg perimeter (Fig. 3.1). Several structures appeared in the head such as the optic capsule and the neurocranium which volume forced reduction of the perivitelline space and increased relative egg gravity at the animal pole (Fig. 3.2). In the opposite pole of the egg, the tail grew in length and reduced in width, being the average width of head and tail of 0.36 ± 0.04 mm and 0.11 ± 0.04 mm, respectively. Such embryonic polarity in body width and density caused an imbalance in the embryo-yolk stability of the prior transient state. The higher gravity of the head pole forced the embryo-yolk region rotate head downwards and consequently the tail shifted to the apical position relative to the dorsal view to regain equilibrium (Fig. 3.2). The upwards shift of the embryo tail positioned it at the vegetal pole making it converge with the apical OD (Fig. 3.3). After the OD-embryo tail physical convergence, the OCL completely entrapped the OD (Fig. 3.4). Noteworthy, primordial OCL cells (pOCL) were already seen near the embryo tail at stages E and F (Fig. 3.2).

**Figure 5:**
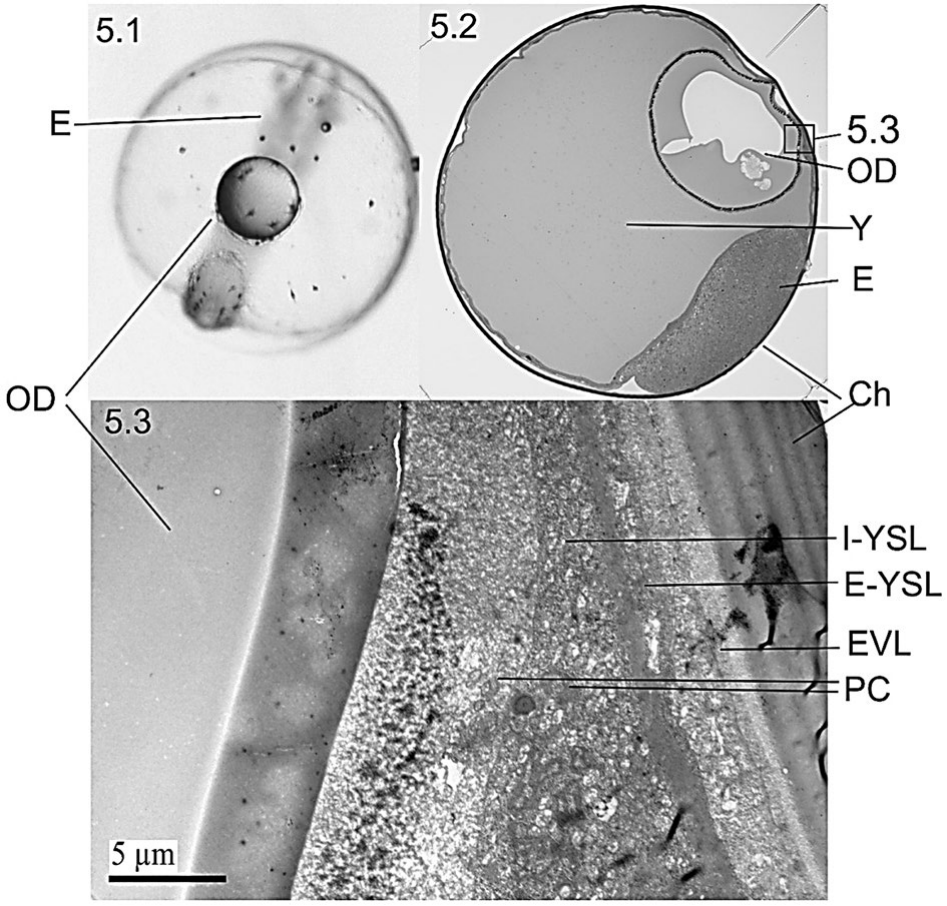
Cellular/tissular structure (**5.1**, **5.2**) and ultrastructure (**5.3**) of the cellular mechanism of adherence of the oil drop (OD) to the oil covering layer (OCL). The OCL is a structure formed in the thickened area of the internal yolk syncytial layer (I-YSL) which primordial germ cells (PC) can be observed before the attachment of the OD to the syncytium (**5.3**). *E-YSL* external yolk syncytial layer, *Ch* chorion, *EVL* enveloping layer, *Y* yolk, *E* embryo.

The microscopic detail showed that the YSL increased in thickness around some star-like primordial structures referred as melanophores/chromatophores^23,31^ (Fig. 4). Those pOCL cells from YSL (termed PC, syncytial precursor cells in Fig. 5) were observed between the OD at internal yolk syncytial layer (I-YSL, Fig. 5.2, 5.3). The connective structures giving rise to the OCL syncytium were the External-YSL (E-YSL), the I-YSL and the enveloping layer (EVL). High electron density images clearly showed that the structural beginning of biosynthesis occurred in the OD periphery (Fig. 5.3).

The OCL developed from I-YSL and partially covered the apical part of the OD (Fig. 6.1). The I-YSL and the OCL formed a continuous syncytium covering the OD simultaneously to the detection of transport vesicles in the yolk (Fig. 6.2). The ultrastructure of the OCL was composed by the endoplasmic reticulum, exocytosis vesicles and mitochondria (Fig. 6.3).

**Figure 6:**
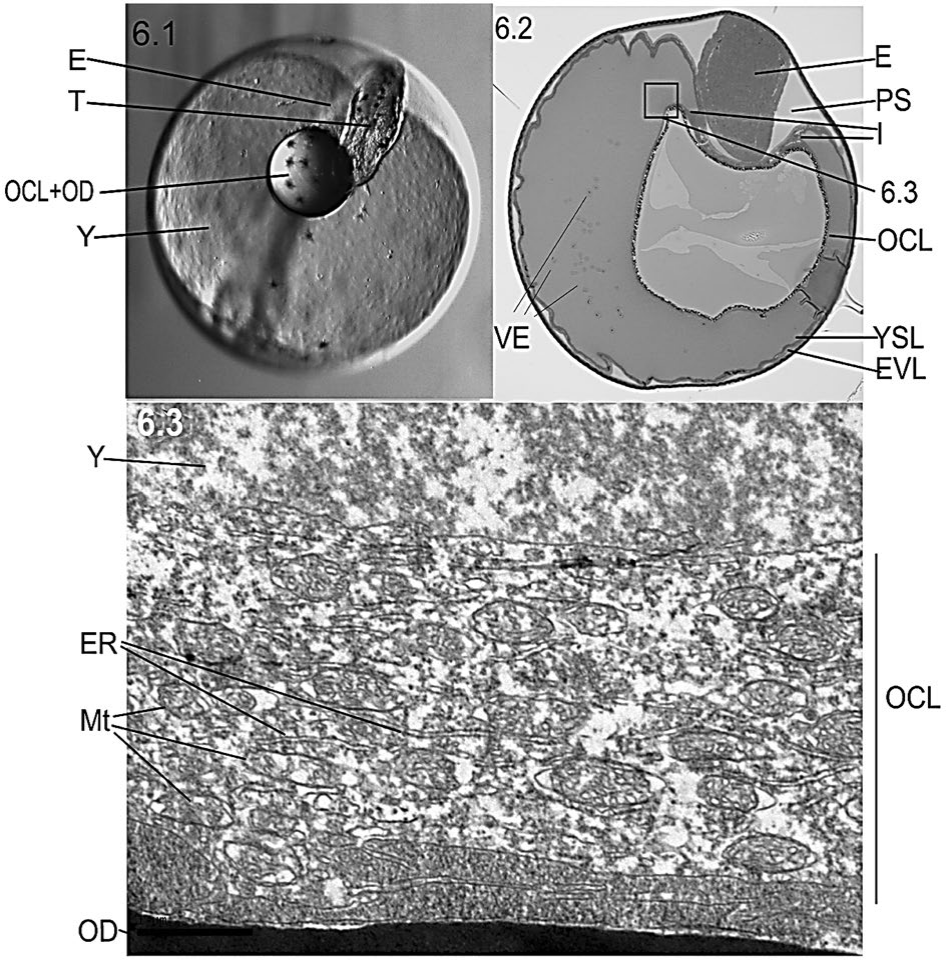
Structure (**6.1**, **6.2**) and ultrastructure (**6.3**) of the cellular mechanism of adherence of the oil covering layer (OCL) to the oil drop (OD) during stage F (54 hpf). In (**6.1**) (photographed 1200×) and (**6.2**) (5 µm-section of the structure shown in (**6.1**)) the OCL traps half the OD to form the complex (OCL + OD). Endoplasmic reticulum (ER) and mitochondria (Mt) are visible organelles embedded in the OCL (**6.3**). *E* embryo, *T* embryo tail, *PS* perivitelline space, *YSL* yolk syncytial layer, *EVL* enveloping layer, *Ch* chorion, *Y* yolk, *VE* vesicles of exocytosis and (I), anchorage grove between OCL and YSL.

**Figure 7:**
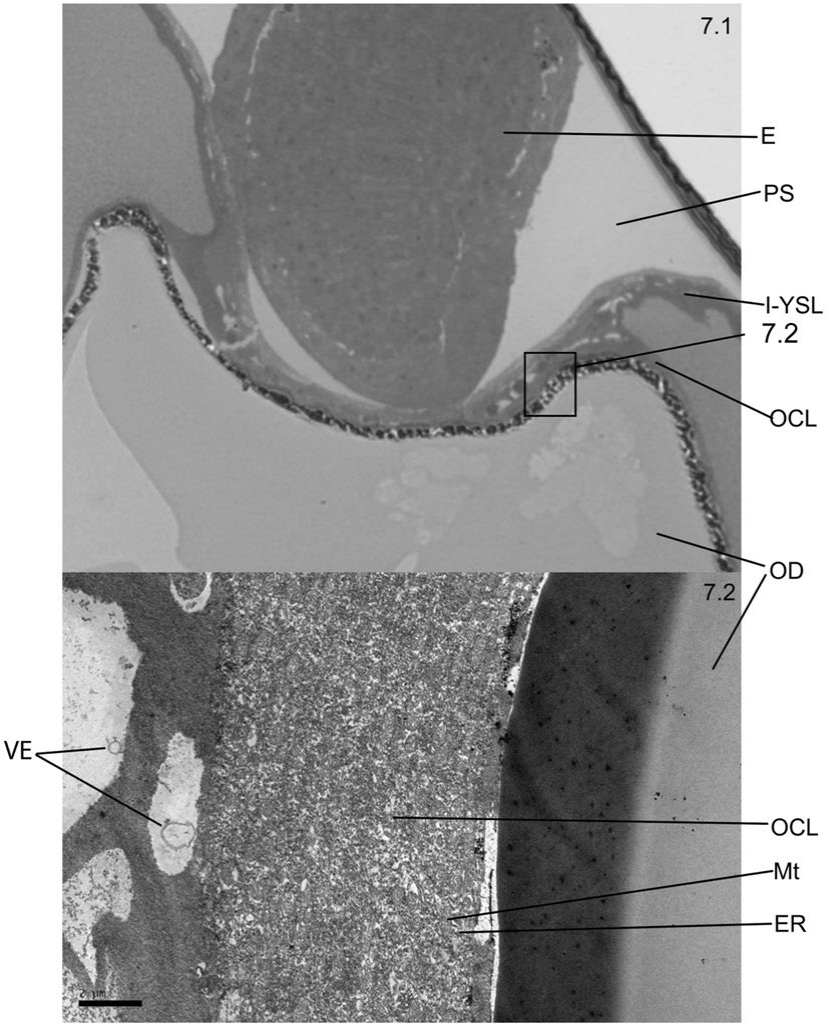
Tissular structure (**7.1**) and ultrastructure (**7.2**) through which part of the exocytosis vesicles (VE) permeate into the embryo (E). *PS* perivitelline space, *I-YSL* internal yolk syncytial layer, *OCL* oil covering layer, *Mt* mitochondria, *ER* endoplasmic reticulum, *OD* oil drop.

The space between the embryo and the OD was occupied by yolk and was invaded by a thickening connective tissue with spaces for passage of molecules (Fig. 7).

Embryos with their OD non-entrapped by the OCL (Oil drop non-adhered, ODNA eggs) exhibited an OD floating in the yolk despite pOCL was observed in the inner part of the embryo tail (Fig. 8.1). In those eggs, the well-developed OCL did not entrap OD (Fig. 8.2) but yolk surrounded the floating OD (Fig. 8.3).

**Figure 8:**
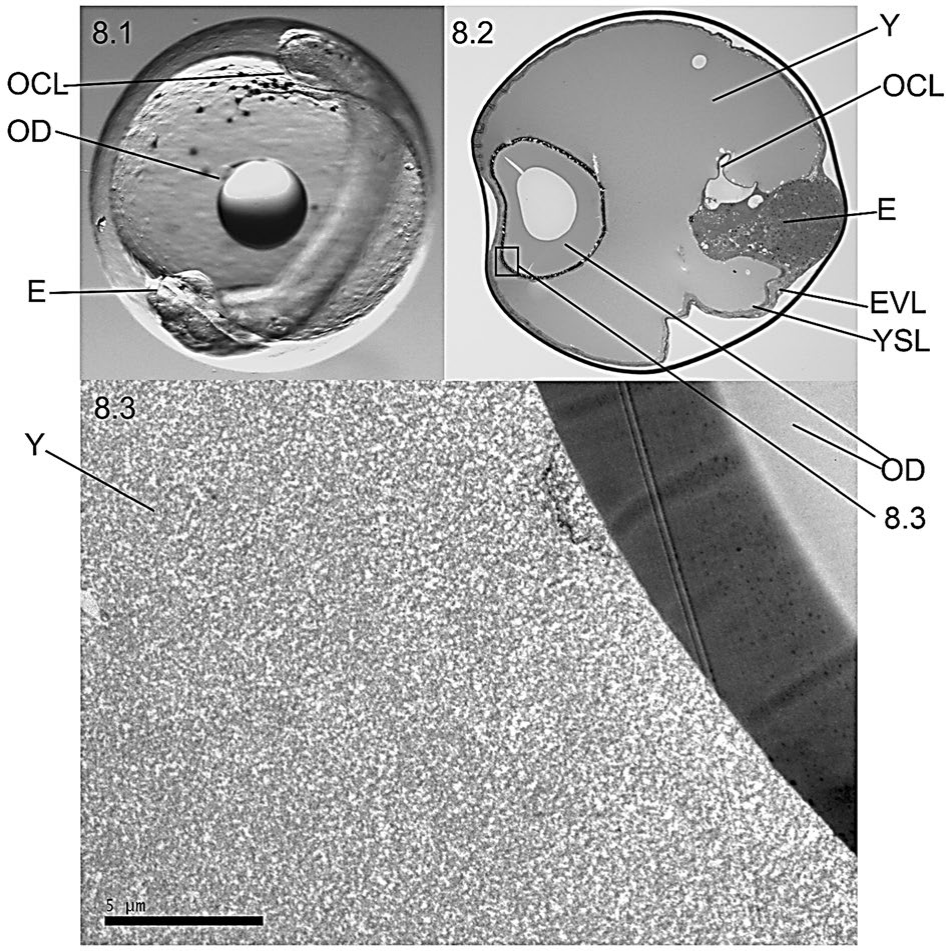
Microscopic tissular structure (**8.1**, **8.2**) and ultrastructure (**8.3**) of a non-adhered oil droplet (ODNA) embryo (E) to the oil covering layer (OCL). The OD floats freely in the yolk (Y) and the OCL is formed in the inner side of the tail regardless the proximity of the OD. *YSL* yolk syncytial layer, *EVL* enveloping layer.

Oil drop (OD)-oil cover layer (OCL) adhered eggs (ODA eggs) continued their development and at stage G (90 hpf) the OD lost mobility due to a complete subjection by the syncytial OCL net (Fig. 9.1). At stages G and H, the embryo occupied the entire egg perimeter with the OD-OCL complex positioned in the middle of the embryo due to its growth in length (Fig. 9.1). The OCL was invariably seen in the middle position of the embryo irrespective of the OD adherence/non-adherence to the OCL (Fig. 9.2).

**Figure 9:**
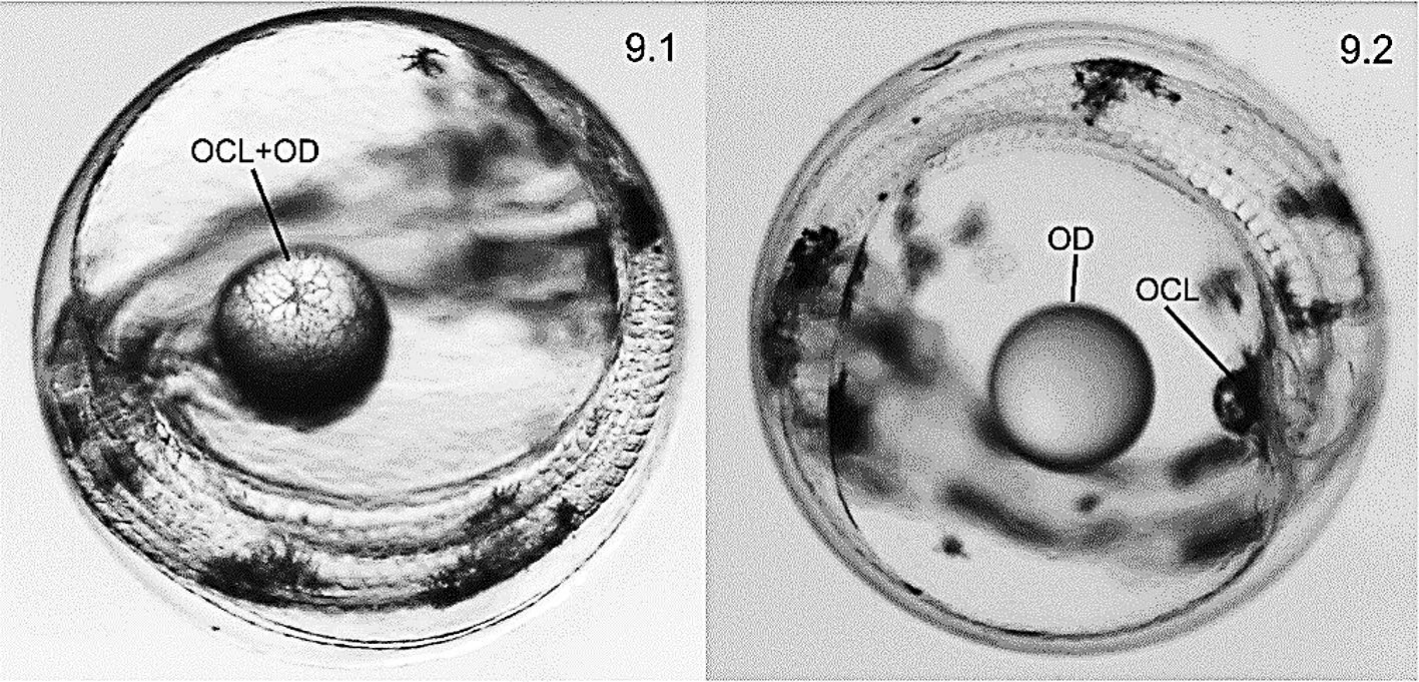
Pre-hatching developmental stages of hake embryos where the oil droplet (OD) is correctly entrapped (ODA embryos) by the oil covering layer (OCL) (**9.1**). Non-adhered OD embryos (ODNA) develop the OCL structure in the inner side of the embryo’s tail irrespective of its distance from the floating OD (**9.2**).

**Figure 10:**
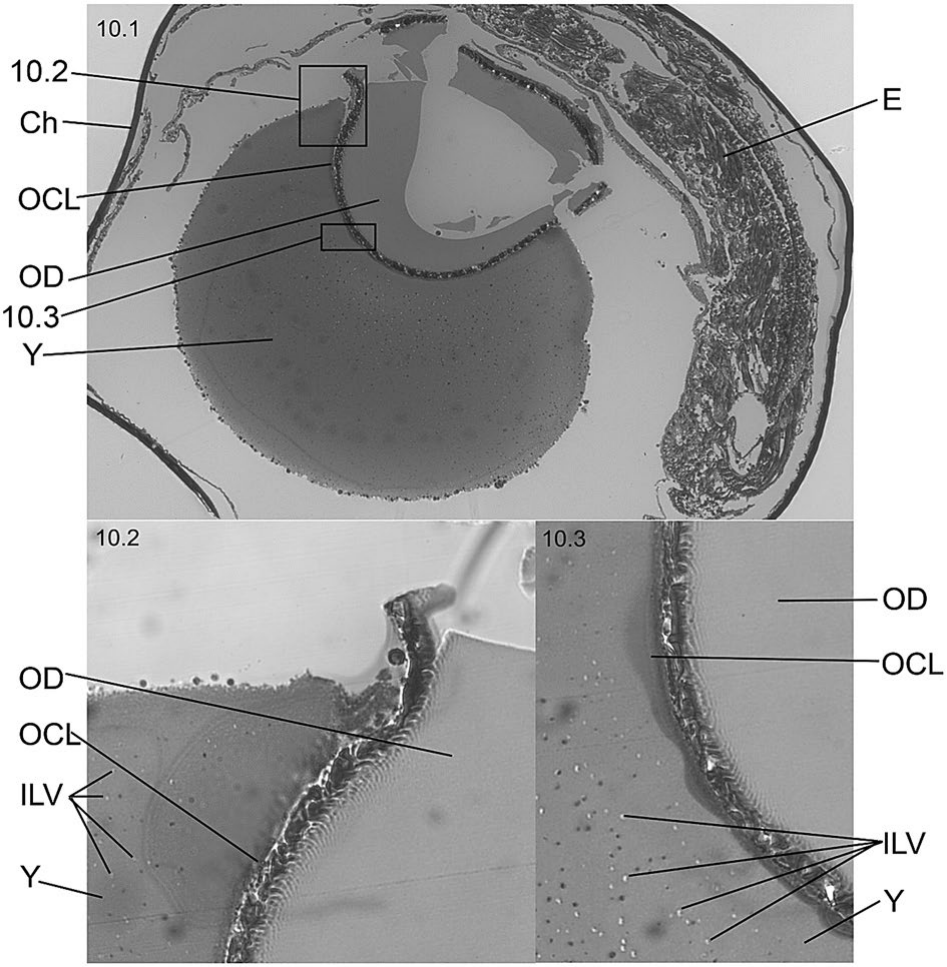
Histological sections of hake embryos at developmental stage G (90 hpf) when the OD-OCL structure transits to the pre-hatching position. *Ch* chorion, *E* embryo, *VE* vesicles of exocytosis, *Y* yolk. (**10.1**) 10× semi-thin subsection (0.5‒1.0 µm) of total egg section; (**10.2**) 40× semi-thin subsection showing the anchorage between OCL, YSL and embryo; (**10.3**) 100× semi-thin subsection showing the diffusion of lipid nutrients from the OD to the yolk from inner OCL lipid vesicles (100×).

In ODA embryos, reserve material spread continuously from the OD and diffused throughout the yolk towards the embryo provided that numerous transport vesicles were observed in the yolk at this phase (e.g., black stains in Fig. 10).

**Figure 11:**
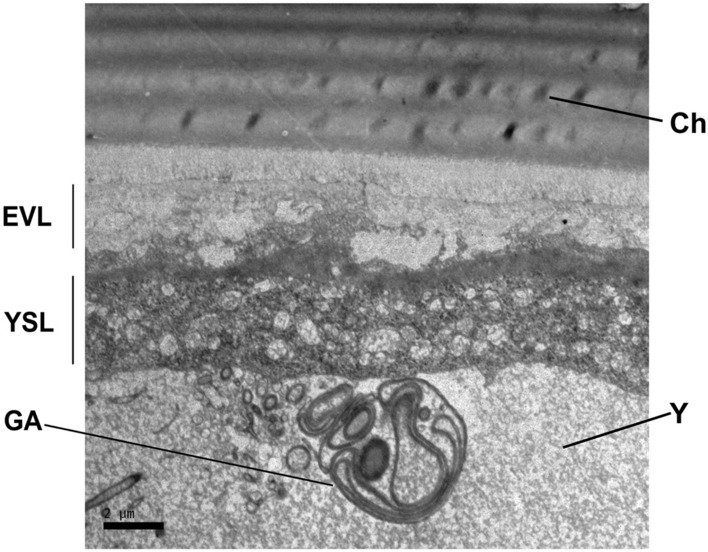
Ultrastructure of the yolk syncytium layer (YSL) where vitelline reserves diffuse from the yolk sac to the embryo by pinocytosis. *GA* Golgi apparatus, *Y* yolk, *YSL* yolk syncytial layer, *Ch* chorion, *EVL* enveloping layer.

Other organelles such as Golgi Apparatus were also patently observed close to the I-YSL (Fig. 11).

### OD-OCL adherence upon egg buoyancy

**Figure 12:**
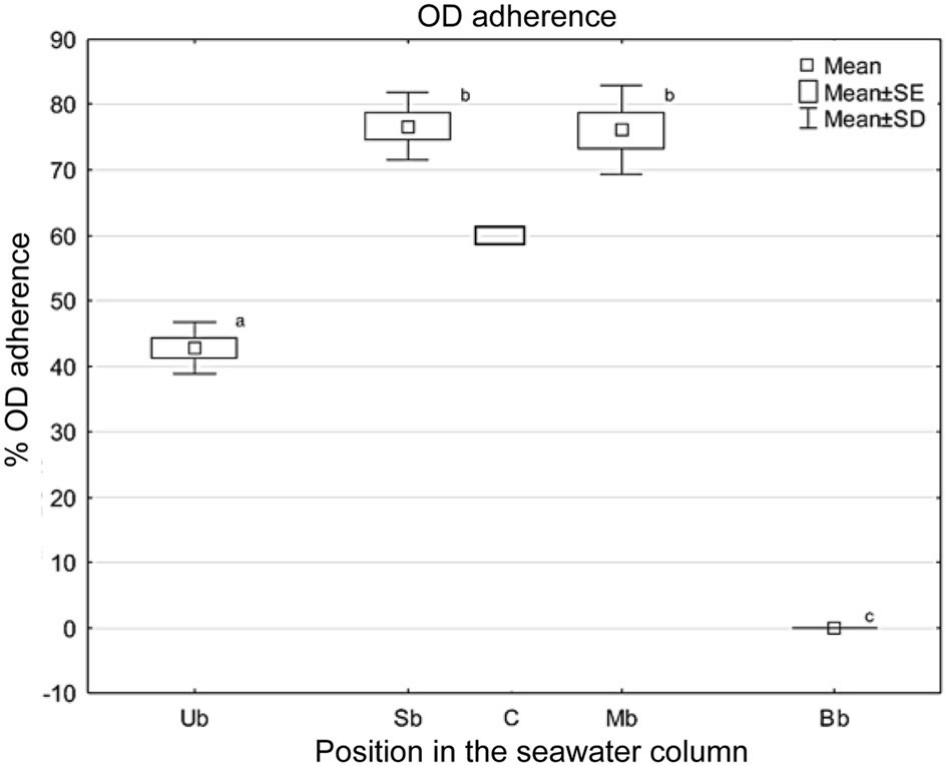
Relationship between buoyancy and percentage of ODA eggs in *M. merluccius*. Eggs distributed at different heights of the water column due to its specific buoyancy. *Ub* ultra-positive buoyancy, eggs are superficially distributed in contact with the air phase, *Sb* positive buoyancy, eggs are immersed just below the surface, *Mb* middle buoyancy, eggs are located in the middle of the water column, *Bb* negative buoyancy, eggs occupy the bottom of the water column, *C* control value from the incubation tank. Bars indicate the standard deviation ODA egg frequency. Significant differences (*p* < 0.05) in the percentage of ODA eggs among buoyancy categories are represented by distinct letters (a, b, and c) on each category.

Oil drop (OD)-oil cover layer (OCL) adhered eggs (ODA) amounted 60% in the 150 L control tank. Buoyancy categories Ub, Sb, Mb and Bb showed a percentage of ODA eggs of 42.80 ± 3.90%, 76.78 ± 5.16%, 76.11 ± 6.80% and 0%, respectively (Fig. 12). Significant differences in the ODA egg percentage were observed among those buoyancy categories (Kruskal–Wallis test, *N* = 24, as 3 burettes, 3 replicates and 4 buoyancy categories, i.e. Ub, Sb, Mb, Bb, *H* = 19.889, *p* = 0.0002). Significant differences in the percentage of ODA eggs were observed between either Ub or Bb and the rest of buoyancy categories (Multiple Comparisons, *Z*-values; *p* < 0.05). No significant differences in percentage of ODA eggs were observed between the buoyancy categories Sb and Mb (Multiple Comparisons, *Z*-value; *p* < 0.05).

### OD-OCL adherence under different seawater supply incubation systems

ODA eggs frequency ranged from 31.3 ± 12.30% (Batch #1) to 90% (Batch #3) (Table 1) but no differences were observed among egg batches (ANOVA, *F* = 0.662, *p* = 0.610). ODA egg frequency ranged from 0% (sprayer system) to 90% (waterfall and traditional systems) (Table 1) and differed significantly among treatments (*F* = 5205, *p* = 0.048) due to the null success of the sprayer system (Tukey-test, *p* = 0.046).

**Table 1:** Percentage of oil drop adherence (ODA) eggs in four European hake egg batches incubated under three seawater supply systems during the embryonic development. Mean ± SD is the average percentage of ODA eggs and its standard deviation per supply system as well as per spawning batch. Distinct superscript letters indicate significant difference of ODA frequency among treatments (one-way ANOVA, *F* = 5.205, *p* = 0.048; Tukey test* p* = 0.046).

Spawning batch (150 mL)	Seawater supply system		
	Sprayer	Waterfall	Traditional
1	–	22.6	40.0
2	0	90.0	90.0
3	–	90.0	90.0
4	0	–	90.0
Mean ± SD	0^a^	67.53 ± 38.91^b^	77.75 ± 25.00^b^

## Discussion

The embryonic development of the European hake is congruent with previous observations of a 96 hpf morphogenetic process from fertilization at 14 °C, comprising VIII developmental stages^32,33^. The observed diameter of artificially fertilized hake eggs in hatchery (diameter 1.071 ± 0.02) also agrees with previous observations (1.06–1.10)^19,32,33,40^ and is similar to that from plankton samples (0.94‒1.06)^18,19,41,42^. Egg diameter differences are believed to be under environmental influence during fertilization as has been reported between Atlantic cod populations from brackish water and marine waters^9,43,44^. The subsequent development is a temperature-dependent process because blastula formation was accomplished in 24 hpf at 14 °C^33^ but in 35 hpf at 12.3 °C or in 22 hpf at 16 °C^40^. Epiboly at 31 hpf, as the first morphogenetic movement in the hake embryo, was characterized by the spreading of blastoderm and YSL over the yolk sac. Hake epiboly was analogous to the one observed in other telolecithal eggs, e.g. zebrafish, where cells dislodge from the animal pole towards the vegetal pole^24^. Hake organogenesis begun at 48 hpf when embryo was already visible in the animal pole. At that time, primordial cells of the oil covering layer (pOCL) appeared at the interface between embryo and yolk, i.e. at the inner syncytium formed by the internal yolk syncytium layer (I-YSL), the external yolk syncytium layer (E-YSL) and the enveloping layer (EVL). The stage E followed epiboly, when embryo was located at the animal pole while yolk and OD situated in the vegetal pole^18^. From there on, the egg specific gravity usually increases due to synthesis of heavy structural proteins employed in embryo growth^5,45^.

By the end of stage F the OCL begun to cover the OD in the vegetal pole and fully entrapped it by stage G. The OCL in hake contained pigmented cells termed melanophores^18^ which might be necessary for the OD lipid protection under the exposed photic area^46^*.* Such melanophores are used as morphological characters for taxonomic identification^18,47^ and current data suggest that those star-like points are in fact primordial oil covering layer cells (pOCL). One of the indispensable functions of the YSL is related with hydrolysis of yolk material and nutrient transport during the embryonic development, i.e. YSL separates yolk and embryo from each other and all endogenous nutrients must trespass it in order to feed the embryonic blastoderm and larval tissues^26,48^. However, here we show that melanophores are only found at OCL but are completely absent from the I-YSL, which suggests that the OCL is a specialized YSL tissue acting as an intermediate OD-YS enveloping zipper as analogous to the thickened vitelline syncytium described in the rockfish *Sebastes schlegeli*^49^.

The pOCL ultrastructure contained both rough and smooth endoplasmic reticulum, numerous mitochondria, and Golgi apparatus, as previously reported from the syncytium membrane of *Pleuronectes platessa*^50^ and turbot *Scophthalmus maximus*^27^. In addition, secretory vesicles responsible for lipoprotein transport from OD to YS were also present. Noteworthy, the endoplasmic reticulum is known to be responsible for the transport of triglycerides and from the current analysis it likely transfers lipids from the OD to the yolk; from here on lipoprotein vesicles went to specific areas of the inner side of I-YSL and entered the bloodstream by endocytosis. Notably, the transport of yolk vesicles in perch (*Perca fluviatilis*) was systematically observed in the inner side of the I-YSL but never in the EVL of the yolk^51^.

Once the stage F was completed, ODA embryos exhibited an OD-OCL complex which was functional until the exogenous feeding. Such structure was responsible for metabolizing and transferring the reserve lipids contained in the OD to the yolk sack^27^, especially just after consumption of yolk and before the exogenous feeding^37^. In ODNA eggs, the OD lipid reserves were not accessible by embryo and larvae^33^ but the pOCL was synthesized anyway close to the embryo tail. This phenomenon denotes a morphogenetic process which falsifies the hypothesis of a non YSL formation as causative of ODNA eggs. This observation in hake eggs is also congruent with those in the gilthead sea bream, *Sparus aurata* where failure of lipid droplet resorption entailed the accumulation of lipoproteins in the endoplasmic reticulum^29^. Moreover, this observation is congruent with a time window < 6 h between stage E and F for the critical assembly of morpho-genetically preformed OD-OCL structures.

Because embryo and yolk are submerged in the perivitelline fluid, the egg content at stage E is in a rotational equilibrium because the animal pole is heavier than the vegetal one (see Fig. 3). During stage E the embryo is distributed uniformly and convexly with minor size differences between head and tail. Embryo at stage E remains in the animal pole opposite to the OD placed at the vegetal pole^18,19^. However, embryo growth during transition between stages E and F entails the development of head structures and somites^18,19,33^ what displaces the egg center of gravity. Such decompensation of the previous equilibrium entails the displacement of the head towards the basal area and the tail to the apical position following the physical laws of bodies immersed in fluids^52^. At this precise time the proliferation of precursor cells near the tail (see Fig. 4) is responsible for the formation of the OCL upon the I-YSL thickening. The apical concurrence of pOCL and the free-floating OD triggers pOCL expansion around the OD.

Abnormal embryo development hampers the approximation of pOCL to OD, inhibiting the positional signal for OCL net extension around the OD. Therefore, egg gravity of an F egg determines its neutral buoyance zone where the perivitelline hydration allows embryo rotating for a successful OD-OCL assembly and therefore the organism viability. On the event that pOCL and OD are not close to each other at the apical pole of the egg at a given developmental timing, the eggs become ODNA and unviable larvae later on. This hypothesis agrees with previous thoughts where “*the dynamics of embryo* + *yolk development is a morphogenetic process subjected to physical forces acting on submerged bodies and determinant for its buoyancy and stability*”^52^ or with that indicated for Norwegian coastal cod “*the osmoregulatory balance in the yolk sac strongly correlates with the achievement of neutral buoyance and hence to assure a correct early egg development*”^45^.

This experiment aimed to study the gravity-dependent buoyancy of a spawning swarm under fixed salinity and temperature and absence of external turbulence. Inner gravity is determined by osmolality changes that occur after the perivitelline space forms between the chorion and the embryonic membrane at fertilization, being filled by ambient seawater^6^. Therefore, the attained volume fraction of the neutral buoyancy in the perivitelline space increases just after fertilization and its specific gravity varies along the development and especially during organogenesis^45^. It can be expected that a single spawning event could exhibit its proper inter-egg variation in gravity and therefore, a particular distribution of egg types upon gravity, i.e. less dense Ub and Sb eggs, isotonic Mb eggs and negative buoyant eggs at the tank bottom.

As expected, the majority of eggs exhibited positive buoyance, i.e. 80% being ultrapositive buoyant (Ub) or positive buoyant (Sb), in consonance with the emergence of hake eggs from the spawning depth at 200 m^18^ to the photic zone at 50 m depth where the highest abundance of larvae are currently found in this species^53^. However, Sb eggs retained below the surface (Sb) and middle buoyant eggs (Mb) showed a 80% ODA success as significantly opposed with egg categories Ub (40% ODA) and Bb (0% ODA). This observation shows that significant differences in ODA/ODNA exist between egg gravity categories distributed in different buoyancy areas and allow to suggest that a relationship may exist between buoyancy and the correct OD-OCL assembly. Likely, extreme buoyancy categories lost their neutral buoyance due to air trapping after emerging to the surface (Ub) or to dehydration followed by sedimentation (Bb)^33^ a status where the embryo loses its rotating capacity to regain equilibrium and properly develop the morphogenetical program of the OD-OCL assembly.

Differences in FAA content/osmolar status have been postulated to explain egg buoyance differences in *Anguilla japonica*^54^ and in *Engraulis ringens*^15^ as well as in *Gadus morhua* between brackish and marine waters^43^. Not only the FAA content as a potential maternally inherited trait^55^ can influence the vertical egg distribution, but also site-specific differential intensity of physical processes has been shown causative of it, e.g. freshwater inputs due to rainfall and rivers^15^. However, under our fixed experimental settings we can exclude physical processes as causative of the vertical hake eggs distribution, as was observed in eggs and larvae of the angelfish *C. aurantonotus* under experimental salinities^56^ or at sea in *Engraulis encrasicolus*^57^. Therefore, we preferentially ascribe the particular egg density to the female condition such as age, size and maternally inherited background which determines the final egg osmoregulatory phenotype^12,45,58^.

The small-scale pilot experiment performed under constant salinity aimed to test the influence of seawater supply systems on the frequency of ODNA eggs. The all-ODNA eggs batches produced by the sprayer indicates that this system does not assure the adequate mixing of hydrophobic hake eggs and air bubbles help eggs rising toward the surface where they eventually die from dehydration^32,36,59^. However, under constant subsurface horizontal flow (the traditional underwater pumping system) or under strong vertical mixing from the surface (waterfall) there are significant higher ODA successes than under the sprayer system. This suggests that the former systems increase the vertical mixing impeding hake hydrofuge eggs to be trapped by the surface tension where dehydration would hamper a correct OD-OCL assembly. Noteworthy, this explanation differs from flow models on hydrodynamic tests where a low flow velocity was insufficient to keep semi-buoyant eggs and larvae in the drift and provoked them to sink to the bottom and perish^60^.

The mechanistic explanation that relates buoyance, supply systems and ODNA eggs is that turbulence or aeration during egg incubation cause an imbalance inside the eggs that leads to internal shifts in the embryo metacenter position. This causes the embryo + yolk rotation to regain stability and balance if and only if such rotation is facilitated by an adequate hydration status of the perivitelline space. As observed in the previous experiment, suboptimally hydrated eggs either become ultrabuoyant or negatively buoyant, and manifest high incidence of OD-OCL assembly failure. While positive egg buoyancy is common in pelagic species^61^ and represents a quality marker in their aquaculture, e.g., *Seriola lalandi*^62^, benthopelagic hake eggs cannot develop successfully in hatchery unless the seawater supply system assures they remain well submerged in the drift until they hatch and develop the ability to swim*.* This scenario probably never occurs under natural conditions because the European hake spawns at ~ 50 to 200 m in depth^23^, where vertical gradients of salinity, bathymetric pressure and temperature exist as to prevent egg layers to fatally reach the surface^63^.

The combination of the oceanic environment and the specific gravity of marine fish eggs determine their optimal buoyance for an effective survival and dispersal. It can be expected that climate variability prompts higher regional seawater temperature, less oxygen concentration, new circulation patterns and lower salinity, all affecting fish reproduction and the bathymetric distribution of early fish stages^64,65^. Several marine female fish have the capacity to adjust the density of eggs prior to ovulation what assure proper buoyancy development and transport for early life stages^45,64^. Therefore, many marine fishes with a plastic spectrum will find their own adaptive way provided those changes are gradual, but would face viability challenges under accelerate change rates^66^. For instance, species with high thermal incubation sensitivity such as the European hake^40^ would be less resilient than those spawning at colder seawaters^67^, e.g. negative buoyancy deformities and low survival have been observed in angelfish *Centropyge aurantonotus* eggs at suboptimal salinities^56^.

Adaptation will depend on how the genetic variability of species face the multifactorial conformation of a novel oceanic environment. Such genetic background consists in maternally inherited biochemical properties determining gravity (e.g. egg lipid, protein and FAA contents, egg size, and chorion thickness)^55,68^. Examples of such plastic response to environmental challenge can be found in anchoveta *Engraulis ringens*^15^, in lobsters *Pleuroncodes monodon*^69^ or in the seatrout *Cynoscion nebulosus*^70^. Interestingly, the recruitment dilemma generated by the lack of correlation between egg production and larval or juvenile abundance^71^ is due to egg development constraints and larvae unviability and likely related to their vertical distribution^72^. Such vertical distribution relates to retention or dispersion during early egg development as reported in western Baltic cod^14^. Irrespective of adult migration later on, aggregation and density at early stages are determinant priors to assure connectivity between spawning and nursery habitats^13,44,57^.

Several research focusses and fishery management criteria are worth considering to better understand the production–recruitment relationship, e.g. enriched spatio temporal knowledge on maternal effects on egg buoyancy^55^, variability in site-specific egg gravity^73^, spatio-temporal distribution of spawners, eggs and larvae^74^ and their genetic effective size^75^, life cycle connectivity between grounds^73,76^, hydrographic modelling^12,77^, etc. would redound on a better assessment of recruitment dynamics and its adaptive potential of exploited species. Importantly in regional modelling studies, priority areas in marine spatial planning which showed higher survival probabilities are not necessarily the fishing areas^12,78^.

If variability of egg gravity phenotypes in a local population has a genetic influence, the more genetic diversity it bears the more resiliently it will face environmental challenges. Phenotypic diversity relates to the demographic structure of populations especially on egg buoyance. For instance, old females of several species seem to produce more buoyant eggs what influences the reproductive capacity of their population and maximizes survival probabilities and connectivity patterns^45^. In this regard, a straightforward management challenge is to hamper the directional fishing on the upper age distribution tail to better support recruitment and genetic diversity facing the global change^79^.

## Methods

### Institutional review board statement

All methods employed in this manuscript are reported in accordance with ARRIVE guidelines (https://arriveguidelines.org). The study was conducted in accordance with the European directive 2010/63/EU and the Spanish legislation on animal welfare (RD53/2013 and RD1386/2018). All the experiments involving fish spawners were approved by the local ethics committee of Centro Oceanográfico de Vigo. Ethical review and approval were waived by the Ethics Committee on Animal Experimentation of the University of Vigo because the analyses on fish eggs fixed in glutaraldehyde did not involve alive animals.

### Broodstock management and egg collection

The European hake broodstock comprised 17 adults caught in Ría de Vigo (NW Spain) and acclimated indoors in 2007^51^. The specimens were maintained in an 8 m^3^ tank equipped with a through-flow seawater system of 500 lx × h^−1^ under natural photoperiod and a faint 80 lx intensity. Seawater temperature and salinity averaged 13.0 ± 1.0 °C and 33.0 ± 1.0 g × L^−1^, respectively. Hakes were fed semi-humid feedstuff composed of fish flour (35%), fish (30%), squid (17%), mussel (18%) and a vitamin premix (6 mg × kg^−1^)^48^. Egg batches outflowing from the tank after spontaneous spawning of the broodstock were systematically trapped in a 500 µm net. Three distinct fertilized egg batches issued from an undetermined number of parents were collected from January 2013 to November 2017. Their embryonic development was monitored to characterize each developmental stage. Egg batches were incubated into 150 L frustoconical tanks under through-flow ultra-filtered seawater at 14.0 ± 0.5 °C, gentle aeration from air stone microbubbles (30–60 µm) and 300‒500 lx of artificial light intensity. Egg development was tracked in a stereomicroscope Leica M8^®^ for timing each embryonic stage and photographed with a Leica IC80^®^HD digital camera. Egg allocation to a given stage followed a reported early-stage classification^40^. Embryo (E) and oil drop (OD) from the final developmental stages, i.e. E to H (Fig. S1 ) were measured using the Leica Application Suite V4^®^ (LAS X) software platform designed for Leica Microsystems Confocal wide field and super-resolution systems.

### Histological and ultrastructure analyses

Several experiments were conducted to analyze the mechanism of oil drop adherence (ODA) to the yolk syncytial layer (YSL) using both, optical and high-resolution histological techniques. Thirty eggs were collected per stage F, G and H (Fig. 1) and classified either as adhered oil drop (ODA) or as non-adhered oil drop (ODNA). Eggs were fixed for 12 h in 5% 0.1 M glutaraldehyde and 0.1 M cacodylate buffer at pH 7.2 followed by successive 12 h washes with buffer 0.1 M cacodylate, pH 7.2. Eggs were immersed in 2% osmium tetroxide then in 0.1 M cacodylate buffer at pH 7.2 for 5 h and then washed twice for 1 h using the same buffer. A final gradual egg dehydration was performed in acetone for 24 h and kept at 4 °C thereafter. A sequential sample impregnation was performed at 4 °C in a mixture of spurr resin (TAAB 812 Resin, Taab Laboratories) and acetone at ratios 1:3, 1:1, and 3:1, successively. Samples were immersed in spurr resin and allowed to polymerize in molds (Beem Capsules) at 60 °C for 48 h. Semi-thin sections (0.5–1.0 µm) were obtained using an ultramicrotome (Leica Reichert ultracuts) and stained with methylene blue on a microscope slide. The histological sections were observed under an Axioskop II Microscope (Carl Zeiss Microscopy) and photographed 10× with an Axiocam Leica HD camera. Ultrathin sections (150–190 nm) of inner embryo areas surrounding OD were analyzed using Transmission Electron Microscopy (JEOL JEM-1010, TEM) and photographed at 1200× and 5000×.

### In vitro egg buoyancy test

The egg buoyancy test aimed to explore the frequency of ODA and ODNA eggs in the seawater column upon their specific buoyancy. Egg density was estimated as the number^33^ of eggs × mL^−1^. Egg sampling was taken from the incubation tank while increasing aeration to assure egg density homogenization. Six thousand eggs were collected and filtered in a 300 μm sieve to remove excess seawater. One thousand eggs per 250 mL filtered seawater were incubated per burette in an isothermal room at 14.0 ± 0.5 °C without aeration. Three burettes, three replicates and 6000 eggs were tested. The remaining eggs in the source batch were maintained in their original 150 L incubation tank and used as control according to the methodology previously described^33^. Eggs distributed in the seawater column during the incubation process upon their specific density. Thirty eggs were collected at stage F, i.e. embryo of 54 hpf (Fig. 1) from each four-seawater column layers, characterized under the stereomicroscope and photographed as described above. Accordingly, eggs were classed as ODA/ODNA eggs from four bathymetric layers: Ub, the ultrapositive buoyance layer, defined as the surface area occupied by an egg of “*hydrofuge nature and tend to float exposing part of its surface above the water level*”^33^; Sb, the positive buoyance layer, where eggs are completely submerged just below the seawater surface; Mb, the middle buoyance layer, containing eggs placed in the middle of the seawater column; Bb, the negative buoyance layer, where eggs are located at the bottom of the burette.

### In vitro turbulence test

The purpose of this experiment was to assess the in vitro influence of the seawater supply system on both, buoyancy and ODNA eggs. Four egg batches from spontaneous spawning events of the broodstock were distributed in 150 L frustoconical incubation tanks using 150 mL egg volume per batch and 860 egg/mL^33^. The putative influence of seawater turbulence on the ratio ODA/ODNA during egg incubation was tested using three seawater supply systems, i.e. waterfall, sprayer, and traditional. The waterfall system consisted on supplying seawater over the incubation tank surface using two 70 cm in length PVC pipes. The pipes were punched 13 times on opposite sides (26 holes per pipe) using a 1 cm diameter drill and placed at 15 cm above the surface of the tank so that seawater was delivered through the holes forming a cascade. The sprayer method consisted on the addition of seawater to the surface of the incubation tank by means of a sprayer attached to the end of a 1.5 cm diameter pipe. The sprayer consisted on a shower head-like device with multiple perforations of 10 µm in diameter. The traditional system consisted on a pipe submerged to the bottom of the tank with ceramic air stones (30–60 µm) providing gentle aeration through microbubbles as commonly used in fish egg incubation of several species^35^. Egg incubation settings were common across treatments, i.e. 1 µm-filtered seawater of salinity 33.0 ± 1.0 g × L^−1^ and 14.0 ± 0.5 °C and natural photoperiod. Thirty post-hatching larvae were randomly sampled from each incubation tank and placed in a Petri dish containing 15 mL of seawater. The final ratio ODA/ODNA was scored in all larvae across treatments using a stereomicroscope LEICA M8^®^.

### Statistical analyses

Parametric tests were applied when arcsin-transformed fertilization rates (*FR*s) was normal or close to normality, i.e. *F*-test, paired *t*-test. Differences in the mean and variance of ODA/ODNA percentage among incubation methods (waterfall, sprayer and traditional) as well as among spawning batches were explored with one-way ANOVA using the software STATISTICA 10.0©. Global significant tests lead to comparison of pairwise means using the Tukey test^80^. When frequency distributions of arcsin-transformed percentages of ODA/ODNA differed significantly from normality and homoscedasticity (Kolmogorov–Smirnov test, Snedecor *F-*test, respectively), the percentage of ODA/ODNA in eggs located at different buoyancy areas were compared using non-parametric statistics such as the one-way ANOVA on ranks test. When significant differences were observed among samples, post-hoc pairwise comparisons were carried out using the Mann–Whitney *U*-test^80^.

### Supplementary Information

Supplementary Figure S1.

## Data Availability

All data generated in this study are included in this published article.
