# Supplementary material for: The embryo-oil drop assembly: the timing and morphology of a critical event for fish early-life history survival
Source: Sci Rep. 2024 Mar 22;14:6918. doi: 10.1038/s41598-024-57429-9 (PMC10959951; doi:10.1038/s41598-024-57429-9)
Supplement: Supplementary file 1 — Supplementary Figure S1. [file 41598_2024_57429_MOESM1_ESM.docx]

Timing the crucial embryo-oil drop assembly: oceanographic facilitation of a precise morphogenetic process

Manuel Nande ^a, b, ✝^, Montse Pérez ^a^, Pablo Presa ^b, *^

Supplementary material

**
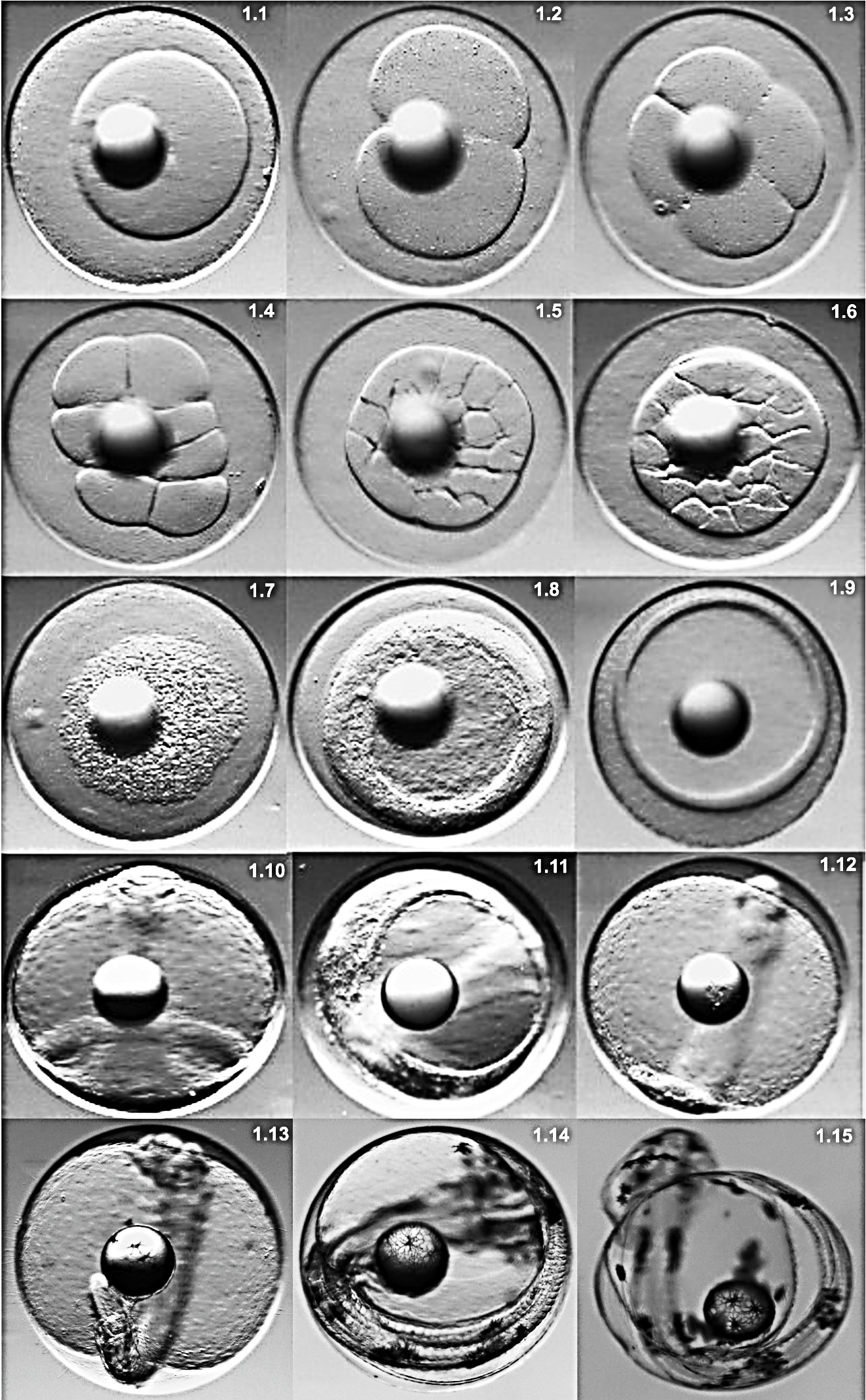
**

**Figure S1.** Relevant stages of the embryonic development of *M. merluccius* eggs prior to the Oil Drop adherence: **1.1.** Fertilized egg (Zygote, 1-cell stage A1); **1.2.** First cleavage (1 hpf, 2-cell stage A2); **1.3.** Second cleavage (4 hpf, 4-cell stage A3); **1.4.** Third cleavage (7 hpf, 8-cell stage A4); **1.5.** Fifth cleavage (10 hpf, 32-cell stage A5); **1.6.** Seventh cleavage (13hpf, 64-cell stage A6); **1.7.** Morula (18 hpf, 512-cell stage B); **1.8.** Blastula (26 hpf, stage C); **1.9.** Gastrula (31 hpf, stage D1); **1.10.** Gastrula (43 hpf, 75% epiboly, stage D2); **1.11.** Gastrula (43 hpf, blastopore, stage D2; **1.12.** Embryo (48 hpf, stage E); **1.13.** Embryo (54 hpf, stage F); **1.14.** Embryo (76 hpf, stage G); **1.15.** Hatching embryo (95 hpf, stage H).
